# Acute Hemorrhagic Edema in Infants: A Continuing Clinical Challenge

**DOI:** 10.7759/cureus.115401

**Published:** 2026-08-29

**Authors:** Hasnae Elhaddadi, Sakout Fatimazahra, Abdeladim Babakhouya, Maria Rkain

**Affiliations:** 1 Department of Pediatrics, Mohammed VI University Hospital, Faculty of Medicine and Pharmacy of Oujda, Mohammed First University, Oujda, MAR

**Keywords:** acute hemorrhagic edema of infancy, children, edema, leukocytoclastic vasculitis cutaneous, pediatrics, purpura

## Abstract

We report a case of acute hemorrhagic edema in a four-month-old boy, which occurred following a viral respiratory infection seven days prior to his admission. The clinical presentation began with the appearance of erythematous macules initially localized to both feet, which then spread to both thighs. All of this occurred against a background of a fever of 38.5 °C, while the child’s general condition remained stable. Initially treated as purpura fulminans, but given the child’s stable general condition and normal laboratory findings, the diagnosis was revised to acute hemorrhagic edema of infancy, characterized by fever and large, coin-shaped purpuric skin lesions, often associated with edema. The differential diagnosis includes hemorrhagic urticaria, other non-infectious vasculitides, erythema multiforme, Kawasaki disease, infectious eczema, and sepsis (meningococcal or otherwise). Some of these conditions carry a risk of mortality. Management is based primarily on symptomatic treatment, with a favorable prognosis.

## Introduction

Acute hemorrhagic edema of infancy (AHEI) is a rare cutaneous leukocytoclastic vasculitis and is possibly underdiagnosed despite having well-defined diagnostic criteria [1]. It was first described over a century ago by Snow in the United States [2]. However, it was not until 1938 that Finkelstein, in Europe, provided the first detailed description of the condition. This is why it is also known as Finkelstein syndrome [3]. Initially considered a variant of Henoch-Schönlein purpura, it is now recognized as a distinct disease. Although many factors have been associated with its etiology, the exact cause remains uncertain. Nevertheless, its benign nature and spontaneous resolution in affected children are well established [4]. Clinically, it is characterized by a triad of fever, coin-shaped purpuric lesions, and edema of the face, ears, and extremities. It generally affects children aged 4 to 24 months; however, cases have been reported in younger children, including during the neonatal period, which makes diagnosis somewhat more difficult [3].

Although the course of the condition is benign and resolves spontaneously, the lesions can be a source of great anxiety for parents and even for medical staff, as they can mimic serious illnesses such as purpura fulminans, meningococcemia, Kawasaki disease, necrotizing fasciitis, toxic dermatitis, erythema multiforme, and even child abuse, among others. The patients’ good overall condition is crucial for diagnosis [5].

We report the case of a four-month-old infant admitted to the pediatric emergency department for purpuric lesions, initially thought to be purpura fulminans, but the diagnosis was revised to acute hemorrhagic edema based on a set of clinical and laboratory criteria. Through this case report, we aim to highlight certain clinical signs that may facilitate the differential diagnosis between AHEI and purpura fulminans, as well as the therapeutic management strategies for clinicians.

## Case presentation

A four-month-old male infant born to non-consanguineous parents presented to the hospital. He had no significant medical or surgical history and no known allergies. He had been exclusively breastfed. His vaccinations were up to date. There was no family history of vasculitis or autoimmune disease. Additionally, the mother reported a history of a nasopharyngeal infection seven days prior. The patient was admitted to the pediatric emergency department for evaluation of macules that had appeared acutely six hours prior to admission, initially localized on both feet and subsequently spreading to both thighs. The patient presented with a fever of 38.9°C but was otherwise in good general condition. He had been given paracetamol at home before being brought to the pediatric emergency department.

Clinically, the patient was alert and responsive with no signs of shock, had a fever of 38.5 °C, a heart rate of 101 beats per minute, an oxygen saturation of 99%, a respiratory rate of 22 breaths per minute, a blood pressure of 96/62 mmHg, and a negative urine dipstick test. Physical examination revealed purpuric macules affecting both feet, legs, and hands, without involving the trunk or face. These lesions were polymorphic, rosette-shaped, and had coalesced on the legs to form purpuric patches. The feet and hands were the site of significant, white, soft edema that did not pit, with preserved pulses. The conjunctival and oral mucosa were unremarkable. Neurological, pleuropulmonary, cardiovascular, abdominal, lymph node, and musculoskeletal examinations were normal (Figures 1, 2).

**Figure 1:**
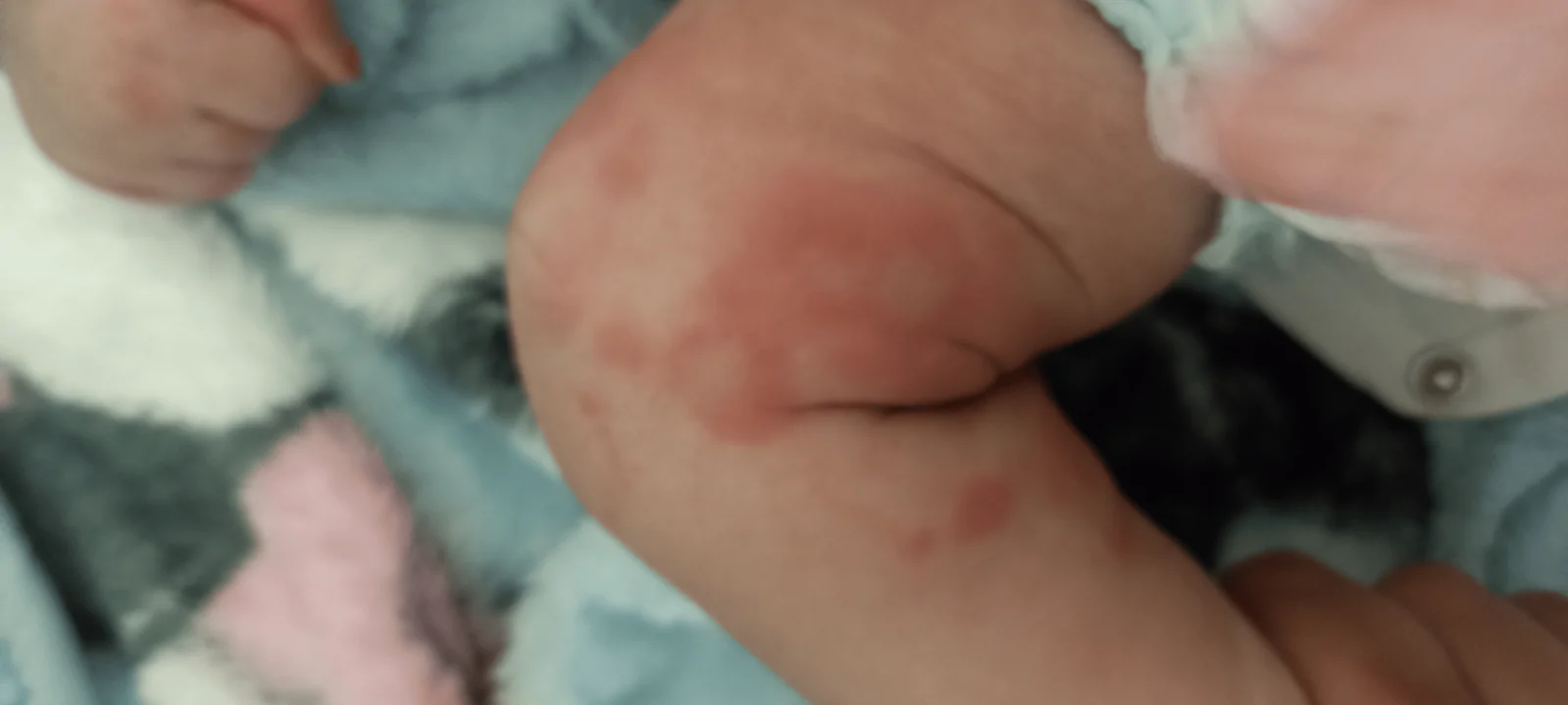
Polymorphic, rosette-shaped lesions that have merged on the thighs and legs to form purpuric patches.

**Figure 2:**
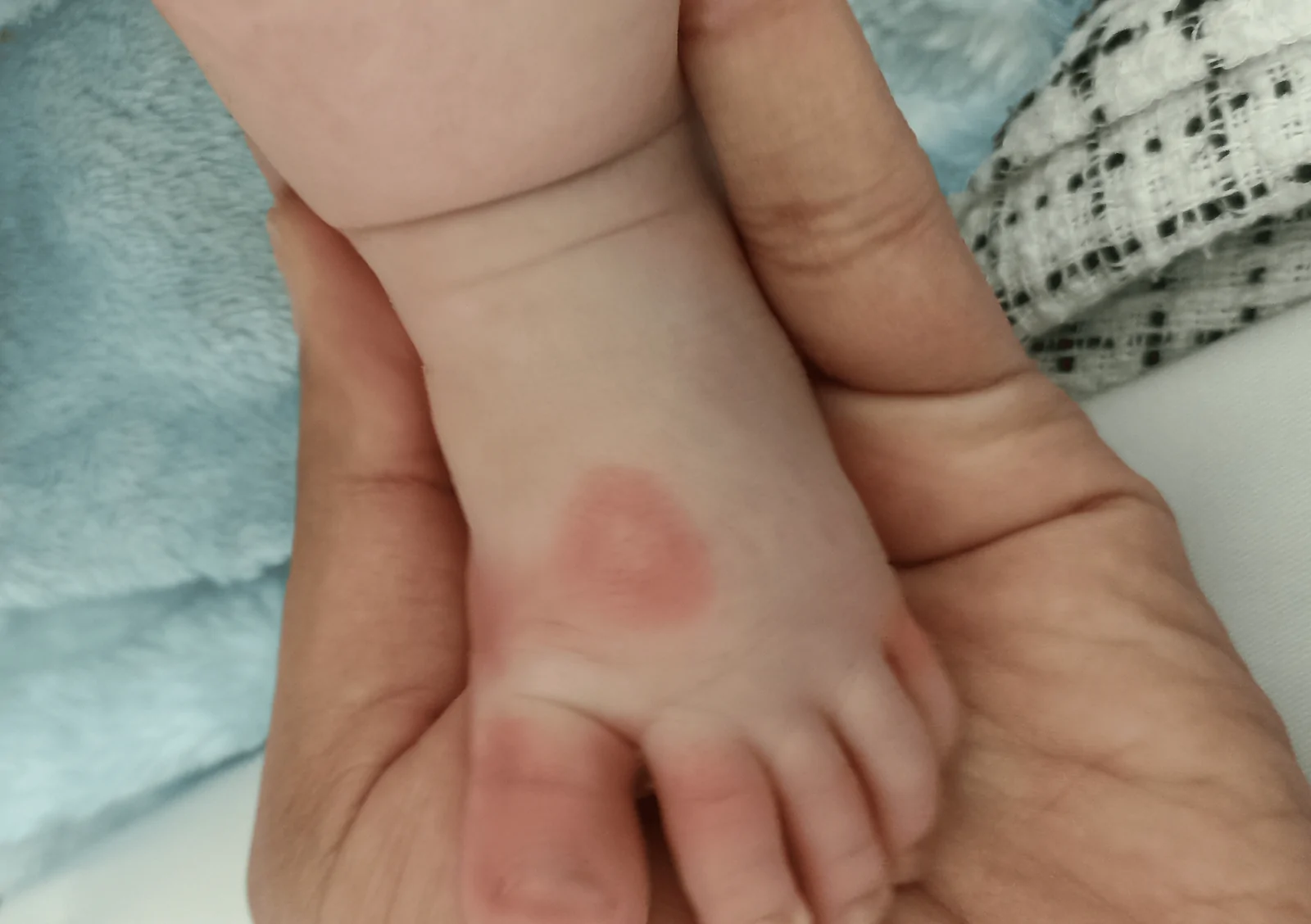
A purplish, rosette-shaped lesion on the back of our patient's foot that does not fade when pressed.

Suspecting meningococcemia, the patient was admitted to the hospital on an emergency basis and managed with extreme caution. Laboratory results showed a C-reactive protein (CRP) level of 2 mg/L, a normal complete blood count with a white blood cell count of 10,600/mm³, a hemoglobin level of 11 g/dL, and a platelet count of 368,000/mm³. Procalcitonin was negative at 0.02 ng/mL. Coagulation tests, renal function, and electrolyte levels were normal. Blood cultures were negative. Analysis of the cerebrospinal fluid obtained via lumbar puncture revealed a clear fluid with a white blood cell count of 0 cells, a direct bacteriological examination showing no abnormalities, normal protein and glucose levels, and a sterile culture (Table 1).

**Table 1:** Patient's biological findings upon admission. SGOT: serum glutamic oxaloacetic transaminase; SGPT: serum glutamic pyruvic transaminase; CSF: cerebrospinal fluid

Laboratory parameter	Values	Reference range
White blood cell (/ul)	10,600	4000 – 10 000
Lymphocytes (/ul)	4700	2000 – 4000
Neutrophil (/ul)	3800	1500 – 7000
Hemoglobin (g/dl)	11	12 - 16
Platelets (/ul)	368,000	150 000 – 450 000
C-reactive protein (mg/l)	2	0 - 5
Prothrombin %	98%	70 – 100
SGOT (UI/l)	22	5 – 35
SGPT (UI/l)	41	5 – 55
Procalcitonin (ng/mL)	0.02	< 0.1
White blood cell in CSF (/mm³)	0	0
Red blood cells in CSF (/mm³)	0	0
Protein in CSF (g/L)	0.20	0.15 - 0.45
Glucose in CSF (g/l)	0.72	0.60 - 0.80
Blood cultures	Negative	

Suspecting Neisseria meningitidis (meningococcal) infection, which remains a diagnostic and therapeutic emergency, empirical treatment with ceftriaxone was initiated immediately. The patient received a single intravenous dose of ceftriaxone (100 mg/kg). No corticosteroids, antihistamines, or nonsteroidal anti-inflammatory drugs were administered. The patient was admitted to the acute care unit for clinical monitoring and remained under observation for 48 hours. Given the distinctive nature of the clinical signs, the patient’s stable general condition, normal laboratory results, and spontaneous clinical improvement, the diagnosis was quickly revised to AHEI. No expansion of the lesions was observed, and the patient remained stable, afebrile, and in good general condition. The outcome was marked by resolution of the edema and purpuric lesions after three days without any treatment. The patient was seen again 48 hours after discharge; his follow-up clinical examination was normal.

## Discussion

AHEI is a rare condition that is often misdiagnosed by most clinicians [6]. Approximately 400 articles have been published in the literature since the disease was first described in 1938. This low number of cases reported in the literature may reflect underdiagnosis, a low incidence of this condition, or confusion with other clinically similar conditions [7].

The pathogenesis of acute hemorrhagic edema in infants remains incompletely understood. However, numerous triggering factors have been reported, particularly infections occurring in the days preceding the onset of lesions. The most commonly described are upper respiratory tract infections (nasopharyngitis, pharyngitis, otitis media, bronchitis), but also conjunctivitis, urinary tract infections, pneumonia, and certain gastrointestinal tract infections. Viral infections, notably those caused by cytomegalovirus, coxsackievirus B4, rotavirus, and herpes viruses, as well as bacterial infections, recent vaccinations, and exposure to certain medications have also been implicated as contributing factors [7-10]. AHEI is considered an immune-complex-mediated vasculitis. In our case, the recent history of a respiratory infection (nasopharyngitis) supports the hypothesis of an infectious trigger. This association is consistent with the literature, which also reports cases following viral respiratory infections or gastrointestinal infections [10].

The diagnosis of AHEI is primarily clinical. When a skin biopsy is performed, histopathological examination reveals small-vessel leukocytoclastic vasculitis, characterized by fibrin deposits in the vascular walls and a perivascular inflammatory infiltrate composed mainly of neutrophils, sometimes accompanied by eosinophils [9]. Deposits of complement (C3) and immunoglobulin A (IgA) may be found in some patients, although they are not essential diagnostic criteria. In our case, since the clinical diagnosis was suggestive, a skin biopsy was not deemed necessary. Nevertheless, this test may be useful in atypical forms or when the differential diagnosis remains unclear [10].

The diagnosis is clinical and is typically based on the following triad of symptoms: fever, edema, and purpura. Typical skin lesions appear as erythematous purpuric lesions-annular, medallion-shaped, or rosette-shaped-that coalesce and merge. These lesions appear rapidly on the face and extremities, sparing the trunk and mucous membranes. Edema generally occurs on the feet, hands, face, and earlobes, and may extend to the scrotum in men [4].

More rarely, epistaxis or hemorrhagic tears have been reported. Extracutaneous involvement is exceptional but may include arthritis, hemorrhagic gastrointestinal manifestations, and scrotal erythema or edema. To study the characteristics of atypical manifestations and the prevalence of recurrences, Rinoldi et al. used a bibliographic database on acute hemorrhagic edema comprising 270 original articles covering 458 individually documented cases (313 boys and 145 girls) published between 1970 and 2019 [11]. Atypical manifestations were observed in 109 of the 458 cases: systemic involvement in 66 cases (61%), atypical rashes in 24 cases (22%), hemolacria in seven cases (6.4%), and a positive family history in nine cases (8.2%). Patients with joint or renal involvement were significantly older than those without atypical manifestations. The duration of illness was three weeks or less in 107 of the 109 cases with atypical manifestations. The diagnosis was confirmed by skin biopsy in 39 cases (36%) of the 108 cases with atypical manifestations [11].

This disorder is benign and generally resolves spontaneously; management is primarily symptomatic. In typical cases, where the patient’s general condition is good and ancillary tests are normal, monitoring and symptomatic treatment are usually sufficient. In cases of clinically suspected sepsis, immediate evaluation and empirical treatment may be necessary until life-threatening causes are ruled out [12], as was the case with our patient.

## Conclusions

AHEI is a benign entity that requires prompt recognition to reassure parents and avoid overdiagnosis. Treatment consists of monitoring and supportive care; in most cases, corticosteroid therapy or antibiotic therapy is not required. In our case, the patient presented with cocarde-like purpura and edema of the extremities; purpura fulminans was initially suspected due to a recent fever reported by the parents, but the diagnosis was quickly revised to AHEI, with a favorable outcome following symptomatic treatment. This case illustrates the importance of a thorough understanding of the typical clinical presentations of AHEI in order to ensure appropriate management and avoid unnecessary invasive tests.
